# Evaluation of a Conference on Cancer-Related Financial and Legal Issues: A Potential Resource to Counter Financial Toxicity

**DOI:** 10.3390/curroncol31050214

**Published:** 2024-05-15

**Authors:** Lauren V. Ghazal, Joanna Doran, Monica Bryant, Brad Zebrack, Margaret I. Liang

**Affiliations:** 1 School of Nursing, University of Rochester, Rochester, NY 14642, USA; 2 Wilmot Cancer Institute, University of Rochester Medical Center, Rochester, NY 14642, USA; 3 Triage Cancer, Chicago, IL 60646, USA; jd@triagecancer.org (J.D.); mb@triagecancer.org (M.B.); 4 School of Social Work, University of Michigan, Ann Arbor, MI 48109, USA; zebrack@umich.edu; 5 Division of Gynecologic Oncology, Department of Obstetrics & Gynecology, Cedars-Sinai Medical Center, Los Angeles, CA 90048, USA; margaret.liang@cshs.org

**Keywords:** cancer, community organization, community resource, financial education, financial toxicity, financial navigation, legal navigation, program evaluation, supportive care

## Abstract

This study describes the conception, development, and growth of the Triage Cancer Conference hosted by Triage Cancer, a national nonprofit organization providing free legal and financial education to the cancer community. We conducted a retrospective analysis of post-conference participant surveys. Descriptive statistics were calculated for participant demographics, and acceptability, feasibility, and appropriateness were evaluated. From 2016–2021, 1239 participants attended the conference and completed post-conference surveys. Participants included social workers (33%), nurses (30%), and cancer patients/survivors (21%), with representation from over 48 states. Among those who reported race, 16% were Black, and 7% were Hispanic. For acceptability, more than 90% of participants felt that the conference content, instructors, and format were suitable and useful. For feasibility, more than 90% of participants felt that the material was useful, with 93–96% reporting that they were likely to share the information and 98% reporting that they would attend another triage cancer event. Appropriateness was also high, with >80–90% reporting that the sessions met the pre-defined objectives. Triage Cancer fills an important gap in mitigating financial toxicity, and formal evaluation of these programs allows us to build evidence of the role and impact of these existing resources. Future research should focus on adding validated patient-reported outcomes, longer-term follow-up, and ensuring inclusion and evaluation of outcome metrics among vulnerable populations.

## 1. Introduction

A cancer diagnosis can often lead to financial toxicity, which has negative and lasting effects on the overall well-being of individuals diagnosed with cancer and their families [1,2,3]. Nearly 50% of cancer survivors experience financial toxicity [4,5], which can be associated with negative effects, including worse physical and mental health, food insecurity [6,7], and even early mortality [8,9,10]. In addition, financial toxicity can have an impact on emotional well-being, which is multi-dimensional and includes the ability to pursue one’s goals [11,12]. For example, patients diagnosed with cancer may value the ability to stay at or return to work not only as a means of maintaining income but also to provide purpose in life, or they may value the ability to provide future financial stability for their loved ones via estate planning. Pisu et al.’s conceptual model depicts how emotion-focused coping (such as accessing social support) as well as problem-focused coping (such as seeking financial assistance, or dealing with insurance) are all critical components of financial toxicity [12]. Thus, the financial toxicity of cancer and its treatment are far reaching as it extends into survivorship and also impacts caregivers [13,14,15].

Nonprofit organizations have grown to offer practical, supportive care services, including direct financial assistance and educational resources around multifaceted components of financial toxicity. With a 17–55% prevalence of food insecurity among cancer survivors [7,16,17], there is a growing recognition of how nutrition and food insecurity factor into the deleterious impact of financial toxicity in cancer care. Moreover, these organizations are the primary referral for many hospital-based financial navigation programs. While there have been increasing calls that multilevel interventions are needed to mitigate financial toxicity [18,19], existing nonprofit resources have not been systematically evaluated.

Often seen as an intervention at the health-system level, financial navigation (or counseling) has emerged as a potential solution, with studies showing its use may significantly mitigate the onset, severity, and duration of financial toxicity among cancer survivors [20,21,22,23]. Unsurprisingly, there has been tremendous growth in these programs as financial-related issues remain a top concern among cancer survivors and caregivers. Financial navigation programs involve helping patients prepare for out-of-pocket medical costs, optimizing health insurance, and maximizing access to financial assistance [21]. In cancer centers lacking or with limited financial navigation services, patients are referred to nonprofit organizations to help provide financial assistance and support. Aside from paying for cancer care, patients must also deal with educational support and work concerns since income and health insurance are frequently tied to employment, navigating benefits such as disability, and estate planning. All these issues may contribute to stressors that negatively impact patients’ and caregivers’ emotional well-being, particularly if patients do not have the appropriate coping mechanisms to deal with these stressors [10].

A challenge to financial navigation is improving the coordination, navigation, and delivery of services [22]. As the cancer survivor population grows and cancer care increasingly shifts to outpatient settings and the home [24], organizations that offer practical, supportive care services, including essential financial resources, education, and support, are more critical than ever [25,26,27]. Cancer care settings face increasingly limited capacity to provide financial resources, including financial navigation services, due to personnel and budgetary constraints [20,21,22]. Thus, organizations are now partnering with academic and medical institutions to identify resources to help patients address financial barriers to oncology care and manage the consequences of financial toxicity [22,28]. While the need to address financial toxicity has increased, even cancer care settings providing financial navigation services are often unfamiliar with the legal and practical issues that contribute to financial toxicity. *Triage Cancer* was founded to provide education on those legal and practical issues [29]. There is also a lack of systematic understanding of the specific resources available and the outcomes generated through the utilization of these services. A better understanding of these data can assist in future efforts to measure the effectiveness of these existing methods to mitigate financial toxicity and tailor them to specific populations. Thus, the purpose of this manuscript is to report on the conception and development of the Triage Cancer Conference and to conduct a program evaluation assessing the program’s reach using participant characteristics and evaluating metrics such as acceptability, feasibility, and appropriateness of the conference among participants.

## 2. Materials and Methods

### 2.1. Triage Cancer

Triage Cancer (https://triagecancer.org/ (accessed on 11 January 2024)) is a national nonprofit organization that provides free education on the legal and practical issues that may impact individuals diagnosed with cancer and their caregivers. It was founded in 2012 by two cancer rights attorneys who believe that the law touches almost every aspect of a cancer diagnosis and that, given the proper information and resources, individuals can use the law as a tool to become their own advocates to access care and avoid financial toxicity. The organization has unique and extensive expertise in developing and delivering educational content on cancer-related legal and financial topics. The organization was established to address the glaring need for clear, reliable information and resources among individuals diagnosed with cancer, caregivers, and health care professionals. Triage Cancer offers educational events and a significant array of educational resources to help people navigate the financial, insurance, employment, and other cancer-related legal and practical issues.

### 2.2. Study Setting, Participants, and Recruitment

The Triage Cancer Conference is a one-day program designed to provide financial and legal education for cancer survivors, caregivers, healthcare professionals, advocates, and others. Initial recruitment efforts began in 2016. Promotion of the conferences was performed via grassroots outreach, word-of-mouth promotion, flyer distribution, electronic newsletters, and social media. Additionally, the event was marketed by professional organizations and community partners (e.g., the Association of Oncology Social Work [AOSW], the Oncology Nursing Society [ONS], and the Leukemia and Lymphoma Society [LLS]). For the years 2016–2019, the conferences were held in specific geographic regions (e.g., Salt Lake City, Philadelphia, Nashville). The locations were selected based on where other national organizations were not hosting in-person educational events, where there were local partners willing to help promote the availability of the events, and where there were underserved communities. Due to the COVID-19 pandemic, the conferences were held virtually in 2020 and 2021.

The Triage Cancer Conferences were free and open to anyone who wanted to attend. Additionally, participants from specific professions were offered free continuing education (CE) credits and contact hours: for years 2016–2020, social workers and nurses were offered CE credits and contact hours, respectively, and starting in 2021, board-certified patient advocates were added. Numerous healthcare industry and nonprofit organization sponsors supported the conference to offset the costs of the venue, meals, and educational materials distributed to participants. All participants were made aware of industry support via a disclosure at the beginning of the conference, and the content that Triage Cancer presents during its educational programs has no relationship to the products or services of these commercial interest organizations.

### 2.3. Session Content

From 2016–2021, a total of six sessions were covered in the conferences (Table 1). Sessions focused on the most common cancer-related topics, including (1) An Introduction to Cancer Advocacy and Managing Finances (later renamed “Cancer Advocacy, Being Empowered, and Introduction to Financial Toxicity”); (2) Health Insurance: Understanding Your Options and Using Your Coverage; (3) Practical Tools for Managing Medical Bills, Your Financial Health, and Estate Planning Documents; (4) Employment 101: Working Through Treatment and Taking Time Off; (5) Employment 201: Disability Insurance (later renamed “Disability Insurance: Options, Applications, and Appeals”); and (6) Cancer Survivorship and Advocacy Opportunities. Session titles were modified throughout the years to capture the increasing awareness of the concept of financial toxicity.

Each session contained two to six learning objectives that were developed by the organization staff consisting of legal experts and oncology professionals. For example, objectives related to the session on health insurance included the goals that participants would be able to “outline various healthcare and health insurance options” and “identify financial assistance options available to pay for health care and other expenses”. In preparation for the conference each year, the Lead Nurse Planner (a PhD-prepared oncology nurse) and the Social Work Consultant reviewed session objectives each year for approval to grant contact hours for nurses and continuing education (CE) credits for social workers.

### 2.4. Measures: Program Evaluation

At the end of the conference, participants were invited to complete a program evaluation survey. For in-person conferences, participants completed evaluations on paper and returned them to program staff before physically leaving the conference setting. For virtual conferences, participants were emailed a SurveyMonkey link that housed the evaluation within 24 h. Participants had seven days to respond to the evaluation. For the in-person conferences, participants were offered one opportunity to provide an evaluation in-person on the day of the event. For virtual conferences, participants received the link in an email following the conference and were sent one follow-up reminder. The program staff maintained attendance records and evaluation data for the conferences throughout the years.

The evaluation survey included several multiple-choice, Likert scale, and open-ended questions. Included were demographic questions, which included race/ethnicity, state of residence, participant identity (e.g., cancer patient or survivor, caregiver, nurse, social worker, attorney, other), and, if self-identified as patient/survivor, the timing in their survivorship (pre-treatment, in-treatment, or post-treatment). Triage Cancer did not collect race/ethnicity data until 2020; thus, there was much missing data to be accounted for. Prior to 2020, there was a conscious effort to eliminate as many barriers to participation in the conferences as possible, including a lengthy registration form. Therefore, demographic questions about race were not included. However, after several years, an assessment was made that collecting more demographic data were useful for several reasons (e.g., illustrating the diversity of audiences helping to perform a gap assessment), and this question was added.

Participants were asked several questions to assess conference acceptability, feasibility, and appropriateness. The acceptability, feasibility, and appropriateness scale (AFAS) is a valid and reliable measure that assesses three perceptual implementation outcomes of training that may be leading indicators of successful training in evidence-based practices [30,31]. In this study, acceptability refers to the extent to which the expectations of the participants about the setting and delivery of the conference and respective sessions were met, including the credibility of the presenters, organization of the conference, and satisfaction with content. Among various stakeholders, acceptability is the perception that a service is satisfactory and agreeable. Feasibility refers to the extent to which participants found the content useful and can incorporate concepts into their own future experiences and daily practices. Appropriateness refers to the extent to which participants found the content relevant to themselves or their patients and addressed critical components leading to financial toxicity [31].

Participants were asked to rate their satisfaction with the components of the conference, which we used to assess the acceptability of the program. This portion of the survey included 14 items, nine of which were on a 4-point Likert scale (1 = strongly disagree to 4 = strongly agree or 1 = poor to 4 = excellent). Item topics included the conference registration process, location (suitability of both online and physical location), duration, content, quality of instructor, the likelihood of attending a future conference, whether the conference and session objectives were met, the perceived value of the information presented at the conference, and whether they would recommend the conference to others. Participants also provided open-ended feedback regarding their overall satisfaction with the session’s content.

The following questions were used to measure feasibility. The overall usefulness of each session was rated on a 4-point Likert scale (1 = poor to 4 = excellent). Participants also provided open-ended feedback regarding what they plan to do with the information gained. We assessed participants’ rating of the usefulness of the sessions as a proxy for their ability to pursue one’s self-defined objectives. For a subset of the participants, emotional well-being was directly discussed in specific sessions (Fostering Resiliency in Families Facing Cancer, The Many Layers of Coping with Cancer). We also captured feasibility data around the extent to which conference participants could incorporate concepts into their own lives (either via experiences as a cancer survivor or caregiver or in their work practice) with questions such as “How [likely/committed] are you to change your behavior or practice based on the information learned today?” and (solely for health care professionals in attendance), “What percentage of your patients are you likely to share this information with?”.

To evaluate the appropriateness, participants were asked for each of the sessions to rate the following statement, “This information will actually benefit myself, my loved-ones, my patients, my community, etc.” on a 4-point Likert Scale (1 = strongly disagree to 4 = strongly agree). Meeting objectives and whether the content was current and/or appropriate were scored on a 4-point Likert scale (1 = strongly disagree to 4 = strongly agree). Participants also provided open-ended feedback on what information they wished they would have received and the kinds of workshops or presentations they would like to see offered in the future.

### 2.5. Data Cleaning and Analysis

We began data cleaning in January 2022. The first author met with program staff at the organization to review data sharing. Program staff in the organization compiled all de-identified evaluation forms in a password-protected folder. For evaluations completed by hand, a second staff member at the organization reviewed manual data entry. To enhance data transparency, rigor, and reproducibility of the methods, we created a codebook for this analysis and for future data analysis with the organization.

We calculated descriptive statistics (e.g., frequencies, percentages, means, and standard deviations) on participant demographic data and variables, assessing acceptability, feasibility, and appropriateness. We also calculated descriptive statistics for participant agreement with the ability of the sessions to meet the pre-defined session objectives. Participants who did not complete any of the evaluation measures were excluded from the analyses. Exemplar quotes were selected to qualitatively illustrate the value that participants found from each of these session topics. We conducted all analyses using Stata.v17. For open-ended responses, Triage Cancer program staff de-identified all data before sharing it with the larger investigative team. These qualitative data were shared in a password-protected folder. Because the data were fully de-identified, and Triage Cancer officials provided written consent for these secondary analyses, this study was deemed exempt from Institutional Review Board review at the University of Michigan.

## 3. Results

### 3.1. Program Participants

From 2016–2021, a total of 2800 participants attended the Triage Cancer Conference. Of these participants, a total of 1239 completed the survey after attending (Response rate = 44.3%). Overall attendance by year grew from 123 participants in 2016 to 644 in 2021. The professional identities among participants spanned the cancer community and were broad-ranging. Participants identified most as social workers (33%) and nurses (30%), with cancer patients/survivors representing 21% of participants. Many participants held more than one identity (e.g., as a cancer survivor and a nurse or a researcher and social worker). Identities classified as administrative, navigator, and supportive care included several job titles (e.g., financial counselor and financial advocates) and are further described in Table 2. The professional identity and role of the researcher included representatives from basic scientists working in pre-clinical oncology drug discovery to health equity and health services researchers.

Out of the total number of participants who were asked to report their race/ethnicity (*n* = 760), nearly 53% were white, 16% were Black, and 7% were Hispanic. Roughly 13% of participants’ racial/ethnic data were missing. Geographic data are depicted in Table 2, which highlights that for the 785 participants who reported their state of residence, 48 states (and the District of Columbia) were represented. Most participants lived in the South (34%) and the Midwest (26%), with 22% in the Northeast and 20% in the West.

### 3.2. Acceptability, Feasibility, and Appointment of Conference and Content

Table 3 displays results from the overall evaluation, specifically highlighting components around the acceptability, feasibility, and appropriateness of the conference and its content. Components were scored positively; across all items, the percentage of participants with responses “agree” or “strongly agree” ranged from 98.01% to 100%. Roughly 99% of participants rated the value of the information presented at the conference and materials provided as “Good” or “Excellent”, and that the conference objective was met. An estimated 98% of participants “agreed” or “strongly agreed” that the content was appropriate for the audience, and 99% “agreed” or “strongly agreed” that the materials were suitable and useful for all attendees. Ninety-eight percent of participants responded that they would attend a Triage Cancer event again. Participants were also “likely” or “very likely” to use another form of Triage Cancer’s service, including webinars (89.6%), the Insurance and Finance Intensive (90.41%), quick guides (95.33%), online resources (96.3%), and educational blogs (82.0%).

Ninety percent or more of participants rated the usefulness of each of the main five sessions as “good” or “excellent”. In the breakout sessions (which had less attendance), due to the nature of the sessions not being offered consistently across the years of analysis, 95% or more of participants rated the sessions as “good” or “excellent”. Breakout sessions on fostering resiliency, post-traumatic growth, and coping with cancer were offered exclusively in 2019. Approximately 86% of participants reported the overall value of the health insurance session as “Excellent”, 84% reported the introduction to cancer advocacy and managing finances session as “Excellent”, and 85% reported the practical tools for managing medical bills, financial health, and estate planning documents as “Excellent”. Participants reported the dissemination of information gained through conferences. Across all sessions again, participants who were health care professionals reported they were likely to share the information that was presented to 75% or more of their patients.

### 3.3. Participant Reports of Meeting Objectives

Table 4 reports the stated educational program objectives for each session, which, overwhelmingly, participants “agreed” or “strongly agreed” were met at least 96% of the time. Exemplar quotes are included in Table 4 that demonstrate the value that participants found from each of these session topics. Participants shared actions that they planned to take as a direct result of attending the conference, which ranged from sharing the information with their network, voting on related legislative policies, feeling empowered to advocate for one’s own medical care by filing for appeals, being more proactive in planning for the future through estate planning, asking for reasonable work accommodations, using practical tips for coping with the psychological and emotional aspects of dealing with a cancer diagnosis, and nutrition planning, among others.

## 4. Discussion

With more psychosocial support for cancer survivors, including financial literacy education, being provided outside of the cancer healthcare setting and by nonprofit organizations, program evaluation is critical to ensuring cancer survivors and their caregivers are receiving evidence-based information and resources. This evaluation of the Triage Cancer Conference demonstrates the wide reach that Triage Cancer has had in terms of recruiting participants in diverse roles and geographic locations. Our data show that surveyed participants had high rates of acceptability, feasibility, and appropriateness, with >80–90% responding favorably to questions within these domains. Critically, there was also high satisfaction (99.4%) with meeting the conference session objectives for topics ranging from health insurance, estate planning, employment, coping with cancer, and how to be an advocate.

This study has important implications for patient, caregiver, and healthcare provider education. Triage Cancer is the longest-running and only free educational conference solely focused on reducing the financial burden and stress through effective navigation of legal and practical issues related to cancer. The high ratings for acceptability, feasibility, appropriateness, and satisfaction with meeting conference objectives are likely due to the concerted efforts Triage Cancer took to involve multiple stakeholders in the development of its program. Medical financial hardship among cancer survivors is complex, and there are multiple levels at which interventions can be targeted, including the patient and family, employer, provider/care team, health care system, state health policy and environment, federal health policy and environment [32]. This analysis allows us to better understand the impact of financial and legal education on key stakeholders at multiple levels. That is, offering Triage Cancer services to not just cancer patients and survivors but also caregivers, health care professionals, employers, human resources professionals, and legal experts aids in implementing a multilevel approach to mitigating financial hardship. Moreover, the model of “educating the educator”, in which education and training are focused on individuals in roles who will have contact with hundreds or thousands of individuals affected by cancer, should further be studied by evaluating the impact of Triage Cancer’s health care professional training, the Insurance and Finance Intensive.

This study also has important implications for using data to inform professional education for financial navigation services in healthcare settings, as there is increasing interest and necessity in increasing the capacity of financial and patient navigation services. As part of these services, nonprofit organizations such as Triage Cancer are commonly used as a referral resource for patients in financial toxicity interventions [33] or in partnering to provide patient and caregiver education [34,35,36,37]). The session objectives represent much-needed topic areas for financial literacy and demonstrate usefulness across healthcare providers, advocates, caregivers, and cancer survivors. In 2019, the National Cancer Institute (NCI) conducted a study to begin to understand the landscape of what financial services were available and some of the challenges to addressing patients’ financial needs as part of cancer care delivery based on that survey [38]. However, despite the widespread availability of financial services, several challenges exist to widespread and effective implementation. These barriers include staff availability, training, and bandwidth, as well as unclear pathways and workflows for identifying individuals experiencing financial hardship and connecting them with services. Other barriers include the administrative challenge of applying for benefits and a lack of cost transparency. In a survey of 221 NCI Community Oncology Research Program (NCORP) practices, 52% of practices referred to outside counseling or case management services, including the American Cancer Society and other patient advocacy groups [39]. Similarly, a survey of National Comprehensive Cancer Network sites reported high rates of referral (62.5%) to third parties [40]. This study provides additional evidence of the importance of nonprofit organization infrastructure in implementing a sustainable model of financial and patient navigation services into the cancer care delivery system.

In a conceptual framework described by Pisu et al., an individual may utilize problem-focused coping or emotion-focused coping to deal with the stressor of the economic burden of disease [10]. These coping mechanisms may have positive or negative downstream effects on patients’ overall emotional well-being, quality of life, or, ultimately, health outcomes. Triage Cancer is also unique in that it addresses coping mechanisms from both domains. For example, Triage Cancer shares problem-focused coping strategies for participants, such as dealing with insurance barriers through filing appeals, using reasonable accommodations to retain employment, or utilizing income replacement benefits like disability insurance. Triage Cancer also provides emotion-focused coping strategies for participants to gain skills, such as building resiliency and accessing specific cancer community resources to enhance personal well-being.

A strength of this study is that the survey data represent responses from different roles and, thus, perspectives. Through qualitative data, we were able to further highlight the impact that the Triage Cancer Conference had on participants, especially specific concrete actions that participants took afterward as a direct result of participating in the conference. Limitations include response bias, as those who responded to the post-conference survey may be those who are already more active individuals or are individuals who were most strongly seeking this type of information in the first place. We are unable to compare the characteristics between responders and non-responders as this was a voluntary survey.

Future research should apply a more empirical, systematic approach. First, validated patient-reported outcome measures should be incorporated and/or developed to evaluate these types of programs. Examples of existing measures that could be applicable include financial distress using Comprehensive Score for Financial Toxicity for patients currently under treatment for cancer [41], InCharge Financial Distress/Financial Well-Being Scale [42], Health Insurance Literacy Measure [43], Patient-Reported Outcomes Measurement Information System (PROMIS) self-efficacy [44], and emotional well-being (i.e., Psychological Well-Being, PROMIS meaning and purpose), and quality of life [45]. Consideration should also be taken of novel metrics, which may not be validated measures but can be measured objectively, such as the number and type of self-advocacy actions taken (e.g., calling my insurance company, applying for a financial assistance program, creating a living will, asked my employer for an accommodation). Importantly, the appropriate metrics for participants may vary based on their roles, such as patient or caregiver vs. health care professional vs. patient advocate. Future evaluations of nonprofit organizations providing financial education and literacy must utilize not only pre- and post-surveys but also incorporate longer-term follow-up assessments to evaluate if the immediate benefits of participation are maintained over an extended period and if participation in these types of events is dose-dependent and can positively influence adherence to recommended care or clinical outcomes among cancer patients and survivors. Additional studies may seek to compare these findings across similar conference programming and apply longitudinal methodologies with conference participation and sustained engagement through program improvement.

Further evaluation of potentially vulnerable populations, such as adolescents and young adults, individuals who do not speak English as their preferred language, immigrants, or older adults, could help to tailor the delivery to the needs of these specific groups. Additionally, household examination of financial toxicity as a result of cancer and its treatment is increasingly recognized; caregivers of cancer survivors experience financial toxicity and experience poor health-related quality of life as a result [13]. Thus, further evaluation of caregivers may also assist in adapting programming. For example, Triage Cancer has already started these efforts by providing specific materials for adolescents and young adults affected by cancer and educational materials and resources in Spanish. Additionally, all materials are free to access for everyone, which includes caregivers, family, friends, and others who desire to learn more about ways to educate themselves and their loved ones about the financial impact of cancer. Participants in different roles or contexts may benefit from differing delivery formats, so continued assessment of acceptability, feasibility, and appropriateness are warranted in addition to evaluating the impact of certain characteristics (e.g., location, format, length) of the conference on the intensity of participation.

## 5. Conclusions

Triage Cancer is a critical resource that provides cancer-related legal and financial education. Current models of cancer care delivery warrant the need to better understand supportive programming on cancer-related legal and financial topics. This paper rigorously analyzes the organization’s programmatic data, which can be used by researchers to inform future multilevel interventions to mitigate financial toxicity. Recognizing that these issues affect not only individuals diagnosed with cancer but individuals with a broad range of chronic or serious medical conditions, Triage Cancer recently launched Triage Health [46]. Thus, the impact of programs like the Triage Cancer Conference has the potential to be exponential and, therefore, deserves attention to further develop this as an evidence-based intervention to address the economic burden of cancer.

## Figures and Tables

**Table 1:** The Triage Cancer Conference Program—Session Breakdown by Year.

Session Title	Year					
	2016	2017	2018	2019	2020	2021
An Introduction to Cancer Advocacy and Managing Finances ^a^	X	X	X	X	X	X
Health Insurance: Understanding Your Options and Using Your Coverage ^b^	X	X	X	X	X	X
Practical Tools for Managing Medical Bills, Your Financial Health, and Estate Planning Documents ^c^	X	X	X	X	X	X
Employment 101: Working Through Treatment and Taking Time Off ^d^	X	X	X	X	X	X
Disability Insurance: Options, Applications, and Appeals ^e^		X	X	X	X	
Breakout Session: Nutrition		X		X		
Breakout Session: Fostering Resiliency in Families Facing Cancer				X		
Breakout Session: Post-traumatic Growth				X		
Breakout Session: The Many Layers of Coping with Cancer				X		
Cancer Survivorship and Advocacy Opportunities					X	

*Note.* Session titles listed above are from the most recent (2021) conference. ^a^ Previously “Cancer Survivorship: What You Need to Know”; “Cancer Survivorship: Advocacy and Being Empowered”; “Cancer Advocacy, Being Empowered, and Introduction to Financial Toxicity”; ^b^ Previously “Health Insurance: How to Get It, Keep It, Use It, and Pay for It”; “Health Insurance: Options, Navigation, and Appeals”; “Health Insurance 101: Understanding Your Options”; “Health Insurance 201: Tips on How to Use Your Coverage”; ^c^ Previously “Managing Finances During and After Treatment”; “Managing Finances and Accessing Financial Assistance Options”; “Be Prepared: Estate Planning and Other Documents”; Managing Finances and Accessing Financial Assistance”; “Managing Finances, Medical Bills and Other Documents You Need”; ^d^ Previously “Employment”; “Returning to Work”; “Employment 101: Understanding Your Options”; “Returning to Work After a Cancer Diagnosis”; ^e^ Previously “Taking Time Off Work and Disability Insurance”; “Employment 201: Disability Insurance”; “Employment 201: Disability Insurance: Options, Applications, and Appeals”.

**Table 2:** Participant Demographics (N = 1239).

	Year						
	2016	2017	2018	2019	2020	2021	Total N
**Number of participants**	99	110	100	169	348	413	1239
**Identity**							**Total N (%) ^a^**
Social worker	19	31	40	32	135	151	408 (32.9)
Nurse	26	37	31	74	78	121	367 (29.6)
Cancer patient/survivor	37	26	17	35	63	85	263 (21.2)
Advocate	16	5	2	8	47	65	143 (11.5)
Caregiver	11	9	10	23	24	43	120 (9.7)
Navigator ^b^	18	2	1	5	19	14	59 (4.8)
Other healthcare worker ^c^	2	4	3	9	8	12	38 (3.1)
Supportive care ^d^	3	3	5	6	9	5	31 (2.5)
Nonprofit professional	2	5	3	2	3	4	19 (1.5)
Attorney	1	0	1	10	0	3	15 (1.2)
Administrative ^e^	1	1	0	0	4	6	12 (1.0)
Researcher	2	1	0	1	5	2	11 (0.9)
Student	1	0	1	0	6	0	8 (0.6)
**Race/Ethnicity ^f^**							**Total N (%)**
White	-	-	-	-	164	238	402 (52.9)
Black	-	-	-	-	44	76	120 (15.8)
Hispanic	-	-	-	-	28	25	53 (7.0)
Asian	-	-	-	-	21	31	52 (6.8)
Prefer not to answer	-	-	-	-	9	9	18 (2.4)
American Indian or Alaska Native	-	-	-	-	5	5	10 (1.3)
Other	-	-	-	-	0	3	3 (0.4)
Middle Eastern and North African	-	-	-	-	0	1	1 (0.1)
Missing	-	-	-	-	77	25	102 (13.4)
**Total**	N/A	N/A	N/A	N/A	271	388	760
**Geographic Region**							**Total N (%)**
South	-	-	-	-	-	-	263 (33.1)
Midwest	-	-	-	-	-	-	203 (25.5)
West	-	-	-	-	-	-	169 (21.3)
Northeast	-	-	-	-	-	-	159 (20.0)
Other (Portugal)	-	-	-	-	-	-	1 (0.1)
**Total**							795

*Note.* ^a^ *n* (%) may not sum to 100 due to missing data or multiple categories selected, for example, role and race. ^b^ Includes financial navigator, financial advocate, financial counselor, state health insurance counselor, certified oncology navigator, and fiduciary. ^c^ Includes medical assistant, physician, advanced practice provider, occupational therapist, physical therapist, nutritionist, dietitian, License and Marriage Family Therapist, pharmacist, psychotherapist, radiation therapist, naturopathic doctor, exercise physiologist, clinical counselor, mammographer. ^d^ Includes community health worker, community outreach, outreach coordinator, outreach education specialist, care coordinator, resource specialist, support group facilitator, patient navigator, ostomy support specialist, certified mastectomy fitter, and fiduciary. ^e^ Includes billing supervisor and program director. ^f^ Race/ethnicity data were not collected at conferences until 2020.

**Table 3:** Overall Conference Evaluation—Acceptability, Feasibility, and Appropriateness (AFAS).

Statement	Overall (N)	Response Options N (%)			AFAS Component ^a^
		Agree	Strongly Agree	N/A	
The online location was suitable	654	106 (16.21)	539 (82.42)		Acceptability
The online facilities were conducive to learning.	668	115 (17.64)	524 (80.37)		Acceptability
Conference objectives were met.	1111	128 (11.52)	976 (87.85)		Acceptability
The course content met my expectations.	669	85 (12.71)	573 (85.65)		Acceptability
The content was appropriate for the intended audience.	667	96 (14.39)	562 (84.26)		Appropriateness
The course content was current.	670	66 (9.85)	602 (89.85)		Appropriateness
Instruction materials were suitable and useful.	659	92 (13.83)	561 (84.36)		Appropriateness
The instructor was responsive to participants (Instructor 1).	669	71 (10.61)	596 (88.96)		Acceptability
The instructor was responsive to participants (Instructor 2).	668	72 (10.78)	594 (88.92)		Acceptability
The instructor was responsive to participants (Instructor 3).	378	33 (8.73)	345 (91.27)		Acceptability
	**Overall (N)**	**Good**	**Excellent**		
Value of information presented at the conference	355	35 (9.86)	317 (89.30)		Acceptability
Value of materials provided	221	17 (7.69)	203 (91.86)		Appropriateness
Usefulness of the session: An Introduction to Cancer Advocacy and Managing Finances	1090	159 (14.59)	914 (83.85)		Feasibility
Usefulness of the session: Health Insurance: Understanding Your Options and Using Your Coverage	1168	151 (12.93)	1009 (86.39)		Feasibility
Usefulness of the session: Practical Tools for Managing Medical Bills, Your Financial Health, and Estate Planning Documents	1106	164 (14.83)	935 (84.54)		Feasibility
Usefulness of the session: Employment 101: Working Through Treatment and Taking Time Off	1052	169 (16.06)	873 (82.98)		Feasibility
Usefulness of the session: Disability Insurance: Options, Applications, and Appeals	871	129 (14.81)	725 (83.24)		Feasibility
Usefulness of the session: Breakout Session: Fostering Resiliency in Families Facing Cancer	36	12 (33.33)	24 (66.67)		Feasibility
Usefulness of the session: Breakout Session: Post-traumatic Growth	29	6 (20.69)	22 (76.86)		Feasibility
Usefulness of the session: Breakout Session: Nutrition	62	18 (29.03)	42 (67.74)		Feasibility
Usefulness of the session: Breakout Session: The Many Layers of Coping with Cancer	13	1 (7.69)	12 (92.31)		Feasibility
Usefulness of the session: Cancer Survivorship and Advocacy Opportunities	285	74 (25.96)	206 (72.28)		Feasibility
Registration Process	450	59 (13.11)	386 (85.78)		Acceptability
Location of conference	288	52 (18.06)	231 (80.21)		Acceptability
Quality of instruction and teaching ability (Instructor 1)	668	49 (7.34)	618 (92.51)		Acceptability
Quality of instruction and teaching ability (Instructor 2).	669	48 (7.17)	621 (92.83)		Acceptability
Quality of instruction and teaching ability (Instructor 3).	375	28 (7.47)	346 (92.27)		Acceptability
	**Overall (N)**	**Too short**	**Just right**	**Too long**	
Length of Conference	947	33 (3.48)	502 (53.01)	412 (43.51)	Acceptability
	**Overall (N)**	**Likely**	**Very likely**	**N/A**	
Likelihood of using Triage Cancer service: Cancer Survivorship Webinars	834	209 (25.06)	538 (64.51)	17 (2.04)	Appropriateness
Likelihood of using Triage Cancer service: Triage Cancer Insurance and Finance Intensive (for health care professionals)	803	181 (22.54)	545 (67.87)	23 (2.86)	Appropriateness
Likelihood of using Triage Cancer service: Quick Guides	836	152 (18.18)	645 (77.15)	14 (1.67)	Appropriateness
Likelihood of using Triage Cancer service: Online Resources	837	143 (17.11)	662 (79.19)	13 (1.56)	Appropriateness
Likelihood of using Triage Cancer service: Educational Blog	827	245 (29.63)	433 (52.36)	15 (1.81)	Appropriateness
	**Overall (N)**	**No**	**Yes**		
Likelihood of attending a future Triage Cancer event ever again	1106	22 (1.99)	1084 (98.01)		Feasibility
	**Overall (N)**	**50%**	**75%**	**100%**	
Percentage of patients likely to share information with from session: An Introduction to Cancer Advocacy and Managing Finances ^b^	665	104 (15.64)	210 (31.58)	315 (47.37)	Feasibility
Percentage of patients likely to share information with from session: Health Insurance: Understanding Your Options and Using Your Coverage ^b^	617	82 (13.29)	172 (27.88)	332 (53.81)	Feasibility
Percentage of patients likely to share information with from session: Practical Tools for Managing Medical Bills, Your Financial Health, and Estate Planning Documents ^b^	590	88 (14.92)	150 (25.42)	327 (55.42)	Feasibility
Percentage of patients likely to share information with from session: Employment 101: Working Through Treatment and Taking Time Off ^b^	582	79 (13.57)	161 (27.66)	304 (52.23)	Feasibility
Percentage of patients likely to share information with from session: Employment 201: Disability Insurance ^b^	584	89 (15.24)	148 (25.34)	304 (52.05)	Feasibility
Percentage of patients likely to share information with from session: Cancer Survivorship and Advocacy Opportunities ^b^	284	44 (15.49)	76 (26.76)	144 (50.70)	Feasibility

*Note.* ^a^ Acceptability refers to the extent to which the expectations of the participants about the setting and delivery of the conference and respective sessions were met; the credibility of the presenters; organization of conference; Satisfaction with content. Sources of data were attendance records and evaluation survey. Feasibility refers to the extent to which participants can incorporate concepts into their own experiences and daily practices. Sources of data were the evaluation survey. Appropriateness refers to the extent to which participants find the content compatible with their own experiences and practices; the relevance of the content to themselves or their patients. Source of data were the evaluation survey. ^b^ These six questions were directed solely to the health care professionals in attendance and referred to the percentage of patients they were likely to share information with from the respective session.

**Table 4:** Ability to Meet Conference Session Objectives.

			Response Options N (%)		
Session	Objective	Overall (*n*)	Agree	Strongly Agree	Exemplar Quotes
An Introduction to Cancer Advocacy and Managing Finances / Cancer Advocacy, Being Empowered, and Introduction to Financial Toxicity	1. Outline how healthcare professionals can engage in various types of advocacy and encourage their patients to be advocates.	1076	154 (14.31)	918 (85.32)	“I plan to help my patients calculate their true health coverage cost and encourage them to get copies of their medical records.” [Advocate] “I plan to [be] sure that everyone I am speaking to is going to vote on the issues that are important to them and their healthcare needs.” [Financial Navigator]
	2. Delineate the major contributing factors to financial toxicity after a cancer diagnosis.	742	91 (12.26)	647 (87.20)	
	3. Articulate ways in which patients can be empowered and engaged in their treatment, including access to clinical trials, second opinions, precision medicine, and genetics.	332	41 (12.35)	289 (87.05)	
	This information will actually benefit myself, my loved ones, my patients, my community, etc.	748	123 (16.44)	617 (82.49)	
Health Insurance: Understanding Your Options and Using Your Coverage	1. Outline the various healthcare and health insurance options available to cancer survivors.	1121	156 (13.92)	958 (85.46)	“Triage Cancer is providing a much-needed service for healthcare workers, patients and families. This was a very clear and thorough introduction to health insurance. This was very empowering to me, because it not only demonstrated just what makes the situation so toxic, but also was the first clear overview of the process I have seen that is also very clear about the different levels of insurance appeal. I also love finally understanding Medicare. Additionally, I especially love the “do the math” comparisons.” [Nurse Practitioner]
	2. Outline practical tools and tips for rebuilding financial health after a cancer diagnosis.	305	31 (10.16)	273 (89.51)	
	3. Identify financial assistance options available to pay for health care and other expenses.	303	30 (9.90)	271 (89.44)	
	4. Articulate how patients can effectively choose between plan options.	633	113 (17.85)	518 (81.83)	
	5. Explain the appeal process.	713	147 (20.62)	560 (78.54)	
	6. Describe consumer protections included in various federal laws.	813	185 (22.76)	623 (76.63)	
	This information will actually benefit myself, my loved ones, my patients, my community, etc.	688	120 (17.44)	558 (81.10)	
Practical Tools for Managing Medical Bills, Your Financial Health, and Estate Planning Documents	1. Outline practical tools and tips for rebuilding financial health after a cancer diagnosis.	966	185 (19.15)	754 (78.05)	“I plan to be more proactive in my health care and its costs” [Nurse] “I plan to use the tools on the site to help me plan any future medical leave so I always protect myself. Additionally, will get my estate docs in order so my husband does not need to stress about those things should anything happen to me!” [Patient with cancer] “I plan to implement these into my practice but also into my personal life—it’s never too early to make plans such as estate planning, medical power of attorney, even if it’s ‘just in case’” [Social Worker]
	2. Identify financial assistance options available to pay for health care and other expenses.	663	111 (16.74)	520 (78.43)	
	3. Articulate the various documents that can make up an estate plan.	920	137 (14.89)	756 (82.17)	
	4. Describe one’s options to protect their rights to make decisions about medical care (e.g., medical decision-making).	552	68 (12.32)	460 (83.33)	
	This information will actually benefit myself, my loved ones, my patients, my community, etc.	653	121 (18.53)	521 (79.79)	
Employment 101: Working Through Treatment and Taking Time Off	1. Describe how the American Disabilities Act (ADA) and Family Medical Leave Act (FMLA) can be useful to individuals diagnosed with cancer	930	127 (13.66)	789 (84.84)	“I liked understanding what protections are in place for individuals and plan to make sure my agency (I am on the leadership team) knows and follows them.” [Social Worker] “I plan to be my own advocate for reasonable accommodations.” [Nurse]
	2. Articulate patients’ disclosure rights and medical exam requirements under various federal and state laws	1016	185 (18.21)	814 (80.12)	
	This information will actually benefit myself, my loved ones, my patients, my community, etc.	634	122 (19.24)	496 (78.23)	
Employment 201: Disability Insurance / Disability Insurance: Options, Applications, and Appeals	1. Articulate how disability insurance can be useful to someone who can no longer work as a result of cancer,	933	151 (16.18)	766 (82.10)	“I liked how you broke down the 12 months retroactive period and backpay—it was helpful to have examples. Additionally, I plan to share the Social Security Disability Insurance (SSDI) timing examples with patients.” [Social Worker] “I plan to use this information (and math!) to help patients make the best decisions to get payment.” [Social Worker]
	2. Describe the legal protections and benefits to which patients and caregivers may be entitled while searching for a job after a diagnosis or while working through treatment.	150	39 (26.0)	111 (74.0)	
	3. Outline practical tools and tips for navigating the job search process, working through treatment, and reasonable accommodations.	150	33 (22.0)	117 (78.0)	
	This information will actually benefit myself, my loved ones, my patients, my community, etc.	627	125 (19.94)	484 (77.19)	
Breakout Session: Fostering Resiliency in Families Facing Cancer	1. Learn about the common reactions of children and teens to a cancer diagnosis in the family and how to identify signs of distress.	31	6 (19.35)	24 (77.42)	“I liked the topic on creativity, it gave me an insight on cancer free zones and creating rituals. Life does not have to evolve solely around cancer.“ [Nurse] “I plan to try to see how our area can tap resources and will try to find resources in our rural area—biggest barrier/challenge to this care.” [Nurse]
	2. Understand the role of resilience in families facing cancer and how to help families facing cancer and how to help families engage their natural strengths to cope with and grow through a cancer diagnosis.	31	6 (19.35)	24 (77.42)	
	3. Explore the world of resources for parents, teens and children affected by cancer, and how to best connect families to valuable support.	29	5 (17.24)	24 (82.76)	
Breakout Session: Post-traumatic Growth	1. Outline post traumatic traits.	20	3 (15.0)	17 (85.0)	“I learned that trauma does not have to crush you but you can learn to grow through it.” [Social Worker]
	2. Articulate the ten ingredients to resiliency.	20	3 (15.0)	17 (85.0)	
Breakout Session: Nutrition	1. Outline common myths about nutrition after cancer.	20	2 (10.0)	18 (90.0)	“I plan to use information to be a better advocate to patients to form better nutritional habits.” [Social Worker] “I plan to work on menu options each week.” [Nurse]
	2. Articulate top nutritional recommendations.	20	2 (10.0)	18 (90.0)	
	3. Describe the role nutrition plays in cancer survivorship.	33	7 (21.21)	25 (75.76)	
	4. Outline steps patients can take to improve their nutrition.	33	6 (18.18)	27 (81.82)	
	5. Identify resources for patients to learn more about improving nutrition.	33	6 (18.18)	26 (78.79)	
Breakout Session: The Many Layers of Coping with Cancer	1. Learn about the common reactions of children and teens to a cancer diagnosis in the family and how to identify signs of distress.	11	1 (9.09)	10 (90.91)	“I learned that there is help available to facilitate how a patient or family members cope with cancer. Additionally, I plan to give my patients and the community some tips to cope with their cancer diagnosis.” [Nurse]
	2. Understand the role of resilience in families facing cancer and how to help families engage their natural strengths to cope with and grow through a cancer diagnosis.	11	1 (9.09)	10 (90.91)	
	3. Explore the world of resources for parents, teens, and children affected by cancer and how to best connect families to valuable support.	11	1 (9.09)	10 (90.91)	
	4. Understand the emotional, physical, mental, spiritual, andfinancial impacts of cancer.	16	4 (25.0)	12 (75.0)	
	5. Learn concepts of post-traumatic growth and resilience and how they relate to cancer diagnosis and can aid recovery and health adjustment	16	4 (25.0)	12 (75.0)	
	6. Learn about community resources to enhance personal well-being	16	4 (25.0)	12 (75.0)	
Cancer Survivorship and Advocacy Opportunities	1. Articulate ways in which patients can be empowered and engaged in their treatment, including access to cancer survivorship care plans.	348	51 (14.66)	286 (82.18)	“I liked that there were concrete examples to get involved and advocate on many levels.” [Social Worker] “Attending really helped me to understand how I can be a better advocate and to manage finances better.” [Social Worker]
	2. Outline various legislative advocacy opportunities that exist to improve the quality of life for those coping with cancer.	285	64 (22.46)	212 (74.39)	
	This information will actually benefit myself, my loved ones, my patients, my community, etc.	283	76 (26.86)	183 (64.66)	

## Data Availability

The datasets generated and analyzed for the current study are available from the corresponding author upon reasonable request.
